# Surgical site infection following minimally invasive lobectomy: Is robotic surgery superior?

**DOI:** 10.1002/cam4.4609

**Published:** 2022-02-23

**Authors:** Yucheng Hou, Yeyan Hu, Weijian Song, Jianfeng Zhang, Qingquan Luo, Qianjun Zhou

**Affiliations:** ^1^ Department of Thoracic Surgery, Shanghai Lung Cancer Center, Shanghai Chest Hospital Shanghai Jiaotong University, School of Medicine Shanghai China

**Keywords:** antibiotic, lobectomy, minimally invasive surgery, robotic, surgical site infection

## Abstract

**Background:**

Surgical site infection (SSI) in thoracic surgery remains a significant cause of morbidity and prolonged hospitalization. Minimally invasive surgery (MIS) has significantly reduced the risk of SSI. We intended to compare whether there was difference between video‐assisted thoracic surgery (VATS) and robotic‐assisted thoracic surgery (RATS) in SSI and highlight possible factors influencing SSI in lobectomy.

**Methods:**

This retrospective study analyzed patients who underwent minimally invasive lobectomy from January 2018 to December 2019. All patients' clinical characteristics and surgery‐related information which may be related to the likelihood of SSI were recorded.

**Results:**

A total of 1231 patients' records were reviewed with 806 VATS and 425 RATS. SSI was classified as deep or superficial SSI. Eighty‐six (7.0%) patients were found to develop an SSI with 62 patients having deep infections and 24 had superficial infection. No statistical difference in the incidence rate and category of SSI was observed between patients undergoing VATS and RATS.

**Conclusions:**

There was no difference in the incidence of SSI between VATS and RATS lobectomy. Male gender, heavy smoking, uncontrolled diabetes mellitus, body mass index (BMI) > 27.9, more blood loss, and the higher National Healthcare Safety Network (NHSN) risk index score (1 or 2) were the independent risk factors of SSI following minimally invasive lobectomy, while male gender, uncontrolled diabetes mellitus, BMI > 27.9, more blood loss and the higher NHSN risk index score (1 or 2) were the main predictors of deep SSI.

## Graphical abstract

There was no difference in the incidence of surgical site infection between VATS and RATS lobectomy. Male gender, heavy smoking, uncontrolled diabetes mellitus, body mass index >27.9, more blood loss and the higher National Healthcare Safety Network risk index score (1 or 2) were the independent risk factors of SSI following minimally invasive lobectomy, while male gender, uncontrolled diabetes mellitus.

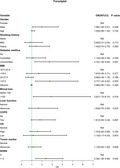

## 1 INTRODUCTION

Infection is the most common postoperative complication. Among them, prevention of surgical site infection (SSI) has always been an important part of surgical technology. It is intuitively that SSI leads to prolonged postoperative hospital stay and an increase in related medical expense.^1^ In severe cases, it can even be life‐threatening. Since 1999, many countries have formulated and updated the guidelines for the prevention of SSI. The key basic measures include aseptic approaches, surgical skills, blood glucose control, maintaining normal body temperature, and appropriate perioperative antibiotics.

**FIGURE 1:**
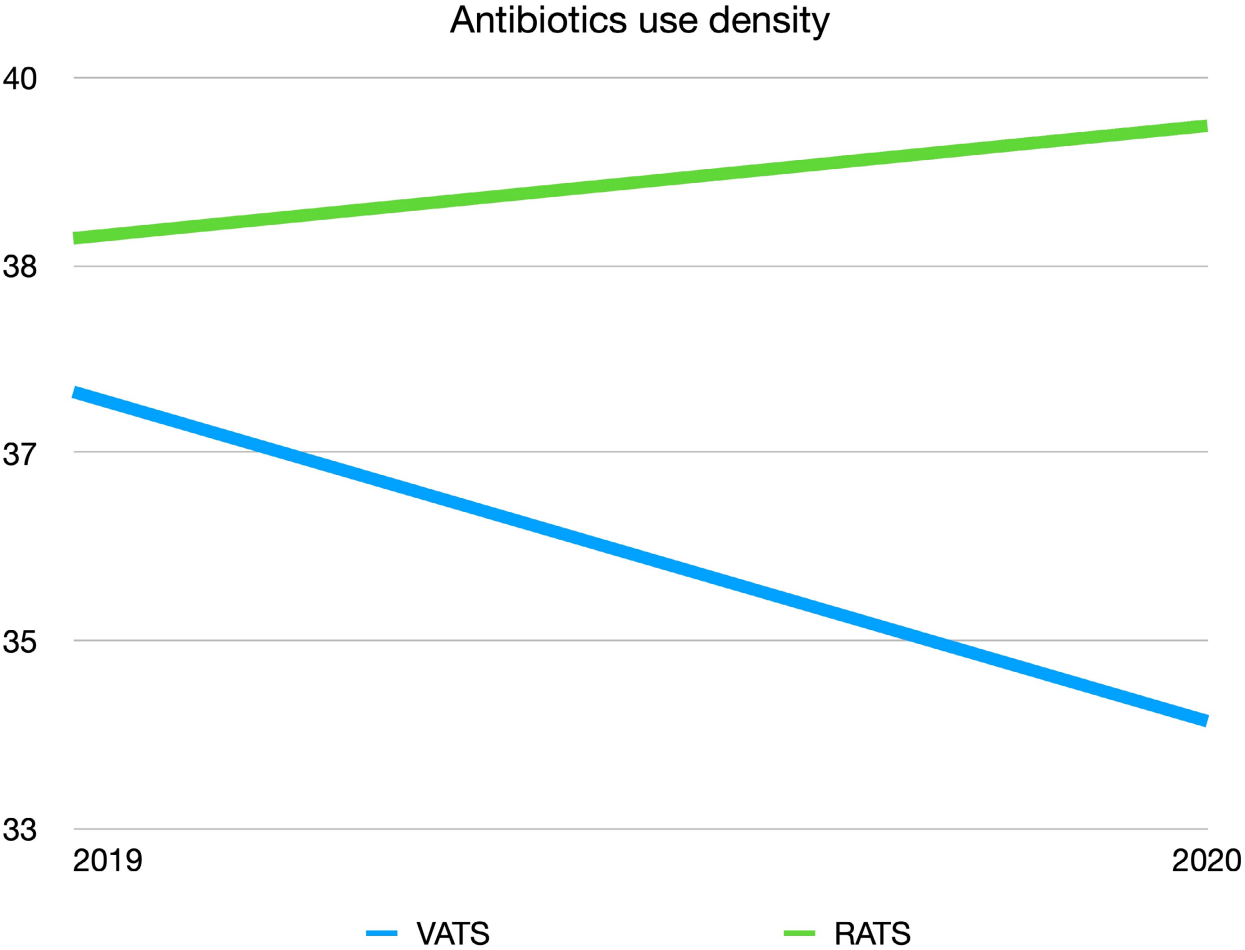
Antibiotics use density, VATS, video‐assisted thoracic surgery; RATS, robotic‐assisted thoracic surgery

At present, surgery is still the main treatment for early‐stage and selected locally advanced non‐small cell lung cancer (NSCLC). With the increasingly complex types of surgery, combined with the long‐term use of hormone, neoadjuvant therapy, previous history of thoracic surgery or trauma, the risk of pulmonary complications can be increased. Many studies have shown that compared with traditional open surgery, minimally invasive surgery (MIS) represented by laparoscopic or thoracoscopic, endoscopic, and robotic‐assisted surgery has significantly reduced the risk of SSI.^2^, ^3^, ^4^, ^5^ However, there is little evidence for this in thoracic surgery. Over the last two decades, robotic‐assisted thoracic surgery (RATS) is changing the concept and mode of MIS in general thoracic surgery practice, especially in pulmonary lobectomy for early‐stage NSCLC. However, high medical costs and lack of haptic feedback are the major limiting factors for robotic‐assisted surgery to become the standard technique of minimally invasive surgery all over the world.^6^ Current studies have shown that RATS has similar perioperative outcomes and comparable oncological outcomes as video‐assisted thoracic surgery (VATS),^7^, ^8^, ^9^, ^10^ while few studies have focused on the incidence of SSI in the two minimally invasive procedures. Based on 2 years of data, antibiotics use density (AUD) of VATS lobectomy was slightly lower than that of RATS lobectomy in our hospital (Figure 1). AUD was expressed as daily dose of antibiotics per 100 patient days (occupied bed days). Therefore, we explored whether there was difference in SSI between RATS and VATS lobectomy, and aimed to determine the related risk factors of SSI following minimally invasive lobectomy.

## 2 PATIENTS AND METHODS

### 2.1 Study design

This retrospective study analyzed patients who underwent pulmonary lobectomy at Shanghai Chest Hospital from January 2018 to December 2019. All patients over 18 years old and less than 90 years old who undergone minimally invasive lobectomy by two certified lung surgeons with at least 5 years of experience in robotic technique and VATS were included. Patients with other resection such as wedge resection, segmentectomy, (bi)lobectomy, bronchial sleeve resection, or pneumonectomy were excluded. Patients undergoing other surgical procedure such as open technique were excluded. Patients who were lost to follow‐up were also excluded.

### 2.2 Data collection

From patients included in the analysis, we collected clinical characteristics and surgery‐related information which may be related to the likelihood of SSI. Patients' clinical characteristics included age, gender, body mass index (BMI), blood type, smoking history, diagnosis, preoperative concentrations of hemoglobin, albumin, diabetes mellitus, existence of hypertension, American Society of Anesthesiologist (ASA) score. Surgery‐related information included surgery side, duration of operation, surgical procedure (VATS or RATS), and blood loss. Duration of operation was reported in minutes from the start of the surgery (incision) to the closing of the skin incision. National Healthcare Safety Network (NHSN) risk index was also recorded which ranged from 0 to 3. As an internationally recognized method for stratifying surgical risk, the NHSN risk index contains duration of operation, surgical wound class, and ASA index. Each variable's cutoff values were a contaminated or dirty surgical incision, an operative duration of 180 minutes and an ASA score of III, with 1 point assigned when each variable exceeded its respective cutoff value.

The main outcome measure was the incidence of SSI within 30 days after operation. According to the Centers for Disease Control and Prevention/National Nosocomial Infections Surveillance guidelines, SSI was defined as an infection that involves skin or subcutaneous tissue (superficial), fascia or muscle layers (deep), or any other anatomic components manipulated during surgery (organ space). In this study, deep and organ/space infection were considered as “deep SSI” because the two are more harmful to health than superficial SSI. Only the first episode of SSIs was included for patients who had more than one SSI during the study period.

All patients underwent perioperative prophylaxis consisting of intravenous administration of 2.0 g Cefazolin Sodium Pentahydrate for injection or 1.5 g cefuroxime within 30 to 60 minutes before the surgical incision, and repeat doses every 12 h for 48 h. Patients with documented –β lactam allergy received clindamycin 0.6 g. If the operation time was more than 3 h, an additional dose of antibiotics was added. All patients had got the same immediate preoperative treatment according to approved skin preparation protocols and use of disinfection solutions.

The follow‐up time was 30 days after the operation. If the patients were discharged, they were followed up by telephone, outpatient, or readmission.

### 2.3 Statistical analysis

The two‐tailed *t* test was used for continuous variables, unless the data were non‐normally distributed. For such cases, we used the Mann–Whitney *U* test for comparison. The *χ* ^*2*^ test or Fisher exact test was used for categorical variables, which were summarized as frequencies and percentages. Independent prognostic factors for SSI were analyzed using univariate and multivariable logistic analysis. All statistical analyses were two‐sided, and a *p* value of 0.05 was considered statistically significant. All statistical analyses were performed using R software (version 3.5.2).

### 2.4 Operative techniques

Our center has more than 10 years of experience in MIS. Robotic technique was introduced in 2009. In all the patients under general anesthesia, double‐lumen tracheal intubation, and isolated‐lung ventilation were performed in the lateral decubitus position.

VATS was performed via three or four incisions. Via a 12‐mm incision, a 30° thoracoscope was inserted in the 6th or 7th intercostal space on the anterior axillary line. A 40‐mm utility incision was performed in the anterior axillary line in the 3th or 4th intercostal space. One 12 mm incision was performed at the 8th intercostal space posterior axillary line. If necessary, a fourth port for assistance was made at the ninth intercostal space on the posterior axillary line. VATS usually needs one surgeon and two assistants, one in charge of the thoracoscope and the other helping retract the lung and exposing the operating fields.

RATS was performed using the da Vinci® robot surgery type Si (Intiuitive Surgical, Sunnyvale, California), and using a three‐port approach with a utility incision. First, a 12‐mm incision for the camera port was made at the 7th or 8th intercostal space posterior axillary line. Second, two 8‐mm port incisions were symmetrically performed at the 7th intercostal space mid‐axillary line and the 9th intercostal space infrascapular line separately. A 40‐mm utility incision was made at the 3th or 4th intercostal space on the anterior axillary line, which used by the bedside assistant for retracting the lung, exposing the operating fields, and stapling and specimen retrieval. Carbon dioxide was insufflated to a pressure of 8–10 mmHg.

## 3 RESULTS

From January 2018 to December 2019, a total of 1231 cases who fulfilled the selection criteria were analyzed.

### 3.1 Population characteristics

The clinical characteristics of all 1231 patients are exhibited in Table 1. The mean age of the entire cohort was 59.14 years (range: 25–82) and the majority of patients were female (713, 57.9%). Around 21.9% of the included patients had a history of smoking. In 369 (30.0%) cases, patients underwent previous operation except thoracic surgery while 25 (2.0%) patients underwent thoracic surgery before this operation. Among the 1231 cases, there were 806 VATS and 425 RATS (65.5% vs. 34.5%, respectively). As reported in Table 1, the two groups had similar demographic characteristics.

**TABLE 1:** Clinical characteristics

Characteristic	VATS (*n* = 806/65.48%)	RATS (*n* = 425/34.52%)	*p* value
Age, median[range]	61 [27–80]	60 [25–82]	0.469
Gender (%)			0.341
Male	347	172	
Female	459	254	
BMI			0.278
<18.5	32	9	
18.5–23.9	459	254	
24–27.9	260	138	
≥28	55	24	
Hypertension			0.513
Yes	572	294	
No	234	131	
Diabetes mellitus			0.590
No	699	361	
Control	64	41	
Uncontrol	43	23	
Smoking history			0.670
Never	629	334	
Mild	53	32	
Heavy	124	59	
Blood type			0.683
A	246	136	
B	220	41	
O	274	115	
AB	66	133	
Previous history of pulmonary surgery			0.123
No	786	420	
Yes	20	5	
Previous operation except pulmonary surgery			0.855
No	563	299	
Yes	243	126	
COPD			0.941
Yes	75	39	
No	731	386	
ASA score			0.667
I‐II	679	362	
III‐IV	127	63	
D‐Dimer			0.646
Normal	698	372	
Abnormal	108	53	
Hemoglobin			0.526
Normal	749	399	
Low	57	26	
Albumin	42.8 ± 3.2	43.0 ± 3.2	0.203
Liver function			0.698
Normal	760	403	
Abnormal	46	22	
Serum creatinine			0.169
Normal	748	406	
Low	35	11	
High	23	8	
Uric Acid			0.075
Normal	695	367	
Low	12	14	
High	99	44	
Tumor marker			0.115
Normal	621	344	
Abnormal	185	81	
Diagnosis			0.482
Benign	66	30	
Malignancy	740	395	

Abbreviations: ASA, American Society of Anesthesiologist; BMI, body mass index; COPD, chronic obstructive pulmonary disease; RATS, robotic‐assisted thoracic surgery; VATS, video‐assisted thoracic surgery.

### 3.2 Perioperative results and surgical site infections

The surgery‐related information and perioperative outcomes of the included patients by approach are listed in Table 2. There was no difference between VATS group and RATS group on surgery site, blood loss, and POD. For patients with SSI, 62 patients developed deep SSI and 24 developed superficial SSI. No statistical difference in the incidence rate and category of SSI was observed between patients undergoing RATS and VATS, while mean operation time varied significantly between the two techniques, of which VATS had a longer operation time (100.7 ± 36.8 minutes vs. 86.1 ± 28.8 minutes, p < 0.001). In addition, there was no difference in the utilization rate of restricted and special antibiotics between the two groups.

**TABLE 2:** Surgery‐related information and perioperative outcomes

Outcomes	VATS (*n* = 806/65.48%)	RATS (*n* = 425/34.52%)	*p* value
Surgery site			0.366
Upper	385	185	
Middle	129	74	
Lower	292	166	
Operation time	100.7 ± 36.8	86.1 ± 28.8	<0.001
Blood loss			0.236
≤100	777	415	
>100	29	10	
SSI			0.888
Non‐SSI	749	396	
Superficial SSI	15	9	
Deep SSI	42	20	
Antibacterial drugs for			0.421
Unrestricted use	703	378	
Restricted use	17	11	
Special use	86	36	
POD (median[range])	4[2–23]	4[2–18]	0.150

Abbreviations: POD, post operation day; RATS, robotic‐assisted thoracic surgery; SSI, surgical site infection; VATS; video‐assisted thoracic surgery.

**FIGURE 2:**
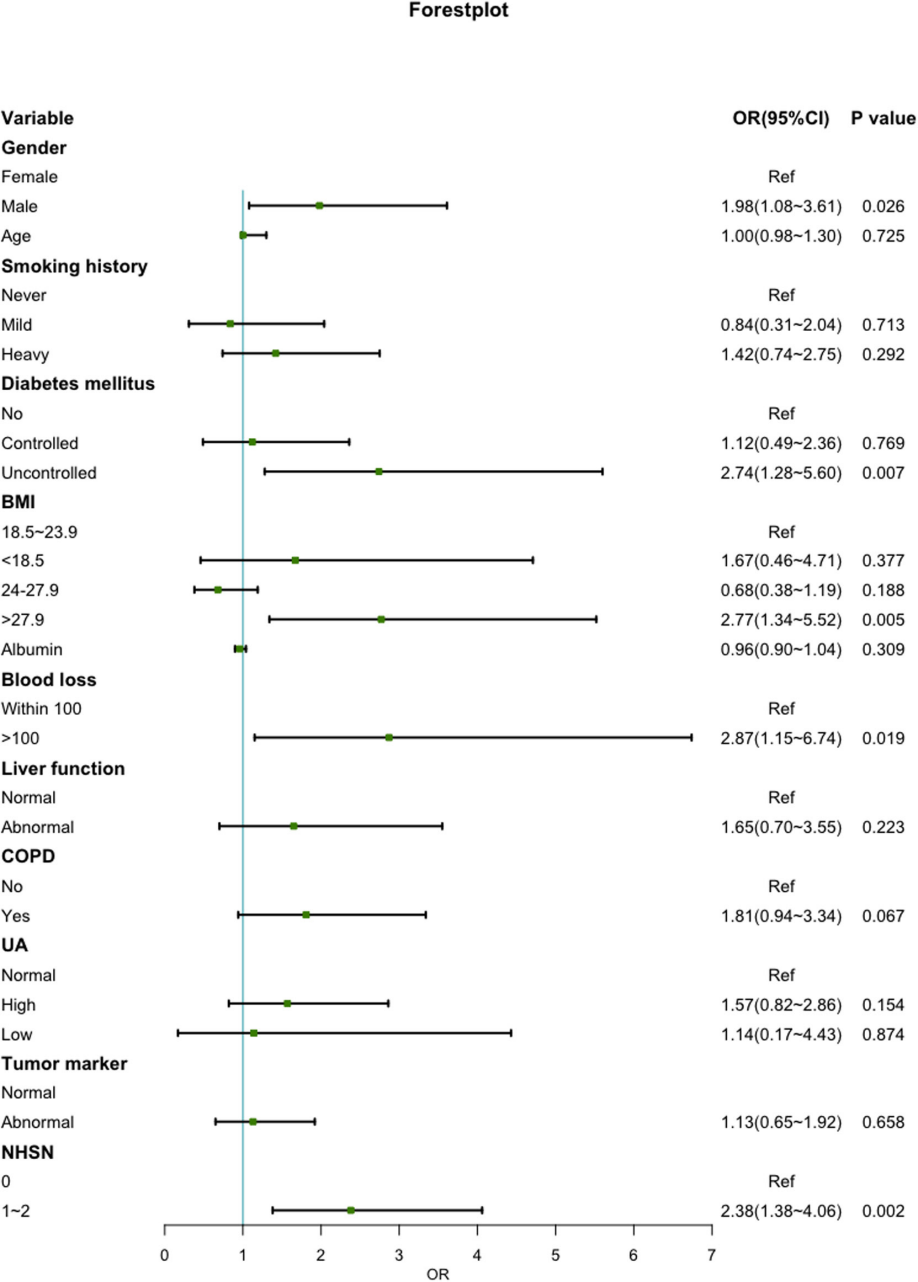
Forest plot illustrating the results of multivariate analysis for risk factors of SSI, OR, odd ratio; CI, confidence interval; BMI, body mass index; COPD, chronic obstructive pulmonary disease; UA, Uric Acid; NHSN, National Healthcare Safety Network

Table 3 highlight the demographic and operative information of the cohort by the presence or absence of SSIs. The logistic regression analysis showed that the risk factors significantly associated with the occurrence of SSI were male gender, elderly, heavy smoking, COPD, ASA III–IV, uncontrolled diabetes mellitus, BMI > 27.9, lower level of albumin, hypemricemia, the longer operation time, more blood loss, and the higher NHSN risk index score (1 0r 2). Furthermore, the multivariate analysis showed in Figure 2 revealed that the independent risk factors of SSI following minimally invasive lobectomy were male gender, heavy smoking, uncontrolled diabetes mellitus, BMI > 27.9, more blood loss, and the higher NHSN risk index score (1 0r 2).

**TABLE 3:** Risk factors for the development of SSI following MIS

Characteristic	SSI	non‐SSI	OR	95% CI	*p* value
Age	62.0[40–79]	60.0[25–82]	1.04	1.02–1.07	<0.001
Gender					<0.001
Male	58	460	Ref		
Female	28	685	3.08	1.94–4.92	
Blood type					
O	32	375	Ref		
A	22	360	0.72	0.41–1.26	0.24
B	25	310	0.95	0.55–1.63	0.84
AB	7	100	0.82	0.35–1.91	0.65
Smoking history					
Never	51	912	Ref		
Mild	28	155	1.60	0.7–3.66	0.26
Heavy	7	78	3.23	1.98–5.28	<0.001
BMI					
18.5–23.9	45	668	Ref		
<18.5	4	37	1.60	0.55–4.7	0.39
24–27.9	21	377	0.83	0.49–1.41	0.48
≥28	16	63	3.77	2.02–7.05	<0.001
COPD					<0.001
No	68	1049	Ref		
Yes	18	96	2.89	1.65–5.06	
Diabetes mellitus					
No	62	998	Ref		
Control	10	95	1.69	0.84–3.41	0.14
Uncontrol	14	52	4.33	2.28–8.25	<0.001
Hypertension					0.11
No	54	812	Ref		
Yes	32	333	1.45	0.92–2.28	
ASA					<0.001
I‐II	54	987	Ref		
III ~ V	32	158	3.70	2.32–5.91	
D‐Dimer					0.22
Normal	71	999	Ref		
Abnormal	15	146	1.45	0.81–2.59	
Hemoglobin					0.72
Normal	81	1067	Ref		
Low	5	78	0.84	0.33–2.14	
Albumin	41.9 ± 4.3	43.0 ± 3.1	0.92	0.87–0.97	<0.001
Liver function					0.01
Normal	76	1087	Ref		
Abnormal	10	58	2.47	1.21–5.02	
Uric Acid					
Normal	66	996	Ref		
Low	2	24	1.26	0.29–5.44	0.76
High	18	125	2.17	1.25–3.78	0.01
Serum creatinine					
Normal	82	1072	Ref		
Low	3	43	0.91	0.28–3	0.88
High	1	30	0.44	0.06–3.23	0.42
Operation time	119.3 ± 47.5	93.9 ± 33.2	1.02	1.01–1.02	<0.001
Blood loss					
≤100	76	1116	Ref		
>100	10	29	5.06	2.38–10.78	<0.001
Tumor marker					0.01
Normal	57	908	Ref		
Abnormal	29	237	1.95	1.22–3.12	
Previous history of pulmonary surgery					0.84
No	84	1122	Ref		
Yes	2	23	1.16	0.27–5.01	
Previous operation except pulmonary surgery					0.50
No	63	799	Ref		
Yes	23	346	0.84	0.51–1.38	
Surgical site					
Upper	46	524	Ref		
Middle	31	427	0.53	0.25–1.1	0.09
Lower	9	194	0.83	0.52–1.33	0.43
Pathology					0.48
Benign	5	91	Ref		
Malignancy	81	1054	1.40	0.55–3.54	
Surgery					0.87
VATS	57	749	Ref		
RATS	29	396	0.96	0.61–1.53	
NHSN risk index					<0.001
0	50	968	Ref		
1–2	36	177	3.94	2.49–6.22	

Abbreviations: ASA, American Society of Anesthesiologist; BMI, body mass index; CI, confidence interval; COPD, chronic obstructive pulmonary disease; MIS, minimally invasive surgery; NHSN, National Healthcare Safety Network; OR, odd ratio; RATS, robotic‐assisted thoracic surgery; SSI, surgical site infection; VATS, video‐assisted thoracic surgery.

In term of deep SSI, Table S1 revealed that male gender, elderly, heavy smoking, COPD, ASA III–IV, uncontrolled diabetes mellitus, BMI > 27.9, hypemricemia, the longer operation time, more blood loss, and the higher NHSN risk index score (1 0r 2) were the risk factors while the multivariate analysis showed in Figure S1 revealed male gender, uncontrolled diabetes, BMI > 27.9, more blood loss, and the higher NHSN risk index score (1 0r 2) were the main predictors of deep SSI.

## 4 DISCUSSION

Despite the recent popularization of minimally invasive approaches, a deeper understanding of the pathogenesis of infections, and routine use of preoperative antibiotics, SSI is still a major cause of morbidity and readmissions for patients undergoing thoracic surgery. SSI is considered as a priority in postoperative infection control that surgeons, nurses, and clinical pharmacists should pay attention to, especially in low‐ and middle‐income countries. Indeed, this problem has not been well monitored, most of which are discovered, judged, and treated by clinical medical staff based on experience. In the field of thoracic surgery, due to the use of cutting stapler, the incision of lobectomy is clean/contaminated without opening the airway. The rate of SSI about clean/contaminated incision is between 5% and 10%. Minimally invasive techniques, which limit size of incision and area of skin contamination, have been shown to reduce the incidence of SSI in thoracic surgery.

We conducted a comparative analysis of SSI between VATS and RATS for lobectomy at a single institution. To the best of our knowledge, this comprehensive observational study is the first to compare the incidence rate of SSI in patients undergoing robot‐assisted and video‐assisted lobectomy. Further, we investigated the risk factors of SSI following minimally invasive lobectomy.

Consistent with previous research results,^11^ male gender was independent risk factors for SSI following minimally invasive lobectomy (*p* = 0.026), which was also risk factors for deep SSI (*p* = 0.012). The impact of gender differences on SSI also occurs in other types of surgical procedures.^12^, ^13^

Taking into account the influence of gender, some scholars believe that males are related to smoking,^14^ and smoking is a risk factor for SSI.^15^ However, this study did not show that smoking was an independent risk factor for SSI after minimally invasive lobectomy.

We also showed that blood loss exceeding 100 ml and the higher NHSN index (1 0r 2) were independent risk factors of SSI, which were consistent with previous studies. The NHSN index contains the wound class, the ASA index, and the operation time. In our cohort, all patients had clean‐contaminated wounds, so the score of this item was consistent, and none of them scored. Patients with higher ASA score (level of III or V), or patients with longer operation time (up to 180 minutes) may be inclined to suffer more risks of SSI following lobectomy.

Obesity is a risk factor for SSI, which has been found in general surgery and orthopedic surgery.^3^, ^16^, ^17^ In our study, BMI > 27.9 was an independent risk factor for SSI, but this conclusion was not reached in deep SSI. We speculate that obesity plays an important role in the occurrence of superficial SSI, which requires a larger cohort to study.

Several studies have found diabetes mellitus is an important risk factor in general and orthopedics surgery.^18^, ^19^, ^20^ However, there is no specific study to discuss the association between SSI and diabetes mellitus after lobectomy. In our study, according to the level of glycated albumin, glycosylated hemoglobin, and perioperative blood glucose, patients with diabetes mellitus were divided into diabetes mellitus controlled group and diabetes mellitus uncontrolled group. Intriguingly, we identified that the uncontrolled diabetes mellitus acted as an independent risk factor of SSI following minimally invasive lobectomy, while controlled diabetes mellitus was not. Chronic hyperglycemia can cause neutrophil dysfunction, resulting in a poor baseline inflammatory response. High levels of perioperative blood glucose may have a synergistic effect. Therefore, continuous monitoring and effective control of perioperative blood glucose levels in patients with diabetes mellitus could alter the risk of SSI.

As is well‐known, VATS and robotic‐assisted technique were becoming cumulatively common worldwide. The evolution of MIS has allowed for smaller incisions with less potential raw surface for exposure to pathogens and remarkably reduced site manipulation compared with open technique. This study demonstrated that the incidence rate of SSI following minimally invasive lobectomy was 7.0%, and there was no difference on SSI between the two surgical technique (VATS 7.1% vs. RATS 6.8%, P = 0.871). Although RATS had a shorter operation time than VATS in our study, the shorter operation time did not bring the benefit of reducing the incidence rate of SSI. Thus, there is no evidence that robotic‐assisted surgical technique can further reduce the incidence of SSI. It is still necessary to verify whether the high cost of robotic surgery justifies its use.

## 5 CONCLUSIONS

There was no difference in the incidence of SSI between VATS and robotic‐assisted lobectomy. Male gender, BMI > 27.9, uncontrolled diabetes mellitus, more blood loss, and the high NHSN index (1 0r 2) were independent predictors of SSI.

## CONFLICT OF INTEREST

The authors have no conflict of interest to declare.

## AUTHOR CONTRIBUTIONS

Yucheng Hou, Yeyan Hu, Weijian Song, and Qianjun Zhou were responsible for the conception and design of the study. Yucheng Hou, Yeyan Hu, Weijian Song, and Jianfeng Zhang were responsible for acquisition and analysis of the data, while Yucheng Hou, Qingquan Luo, and Qianjun Zhou were in charge of statistical analysis. Yucheng Hou, Yeyan Hu, and Weijian Song drafted the manuscript. Qianjun Zhou and Qingquan Luo revised and commented on the draft and all authors read and approved the final manuscript.

## ETHICS APPROVAL AND CONSENT TO PARTICIPATE

The study was approved by the Ethics Committee of Shanghai Chest Hospital (No. KS2011). Written informed consent was obtained from all patients.

## Supporting information


Supplementary TableS1

Supplementary FigureS1

## Data Availability

The data that support the findings of this study are available from the corresponding author upon reasonable request.
