# Supplementary figures and images for: Surgical site infection following minimally invasive lobectomy: Is robotic surgery superior?
Source: Cancer Med. 2022 Feb 23;11(11):2233–43. doi: 10.1002/cam4.4609 (PMC9160803; doi:10.1002/cam4.4609)

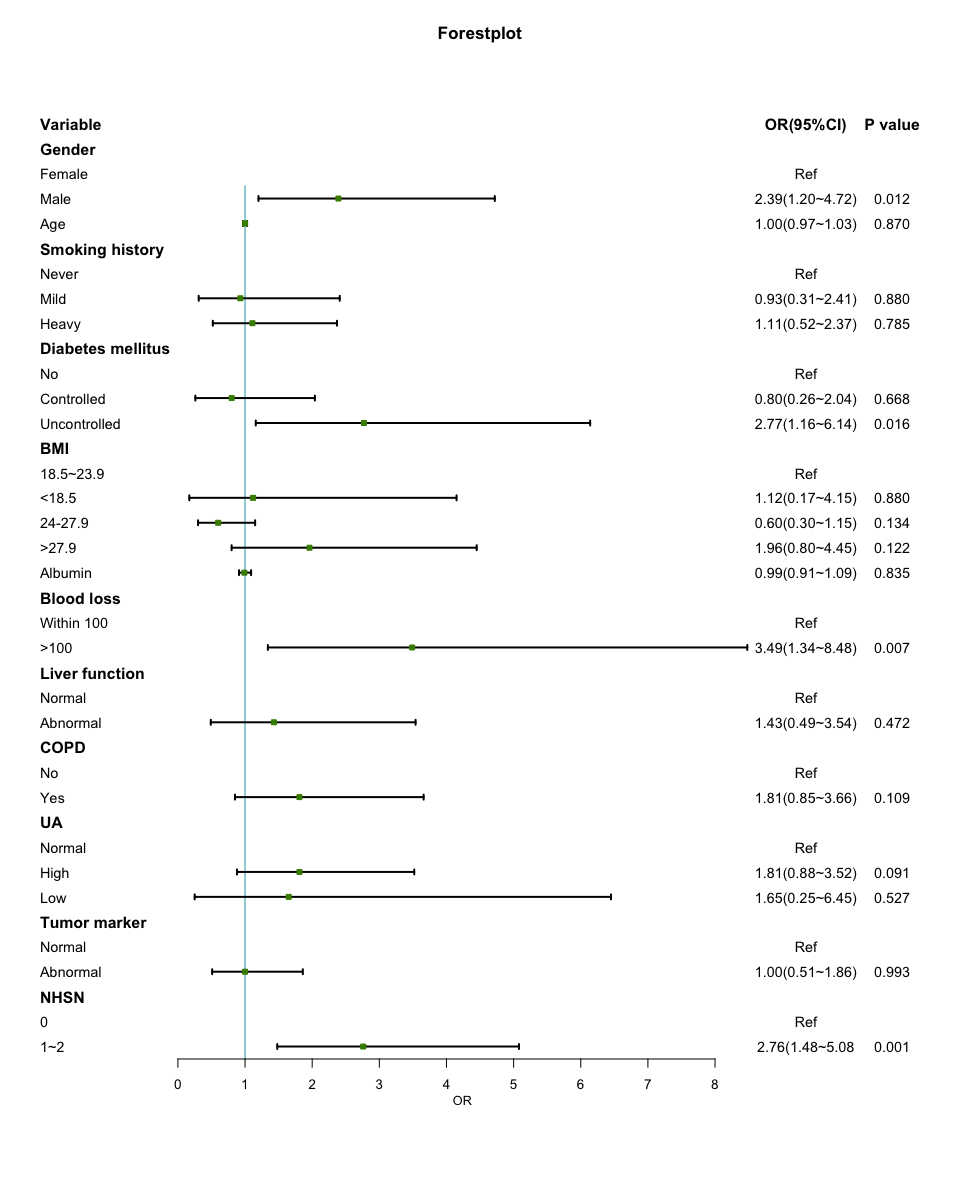

Supplement: Supplementary file 1 — Supplementary TableS1 [file CAM4-11-2233-s001.tiff]
