# Supplementary material for: Surgical site infection following minimally invasive lobectomy: Is robotic surgery superior?
Source: Cancer Med. 2022 Feb 23;11(11):2233–43. doi: 10.1002/cam4.4609 (PMC9160803; doi:10.1002/cam4.4609)
Supplement: Supplementary file 2 — Supplementary FigureS1 [file CAM4-11-2233-s002.docx]

Supplementary Table 1 Risk factors for the development of deep SSI following MIS

| Characteristic | Deep SSI | non-SSI | OR | 95%CI | P value |
| --- | --- | --- | --- | --- | --- |
| Age | 62.0[40-79] | 60.0[25-82] | 1.05 | 1.00-1.10 | 0.03 |
| Gender  Male  Female | 42  20 | 460  685 | Ref  2.98 | 1.26-7.02 | 0.01 |
| Blood type  O  A  B  AB | 24  17  17  4 | 375  360  310  100 | Ref  0.65  1.21  1.41 | 0.21-2.01  0.45-3.26  0.37-5.40 | 0.46  0.71  0.62 |
| Smoking history  Never  Mild  Heavy | 39  6  17 | 912  155  78 | Ref  0.97  5.39 | 0.13-7.59  2.34-12.44 | 0.98  <0.001 |
| BMI  18.5-23.9  <18.5  24-27.9  ≥28 | 36  2  15  9 | 668  37  377  63 | Ref  4.01  1.18  8.25 | 0.84-19.24  0.42-3.24  2.97-22.89 | 0.08  0.75  <0.001 |
| COPD  No  Yes | 49  13 | 1049  96 | Ref  2.88 | 1.05-7.87 | 0.04 |
| Diabetes mellitus  No  Control  Uncontrol | 47  5  10 | 998  95  52 | Ref  3.50  5.12 | 1.25-9.85  1.64-15.96 | 0.02  <0.001 |
| Hypertension  No  Yes | 40  22 | 812  333 | Ref  1.34 | 0.78-2.29 | 0.28 |
| ASA  Ⅰ-Ⅱ  Ⅲ~V | 39  23 | 987  158 | Ref  3.68 | 2.14-6.33 | <0.001 |
| D-Dimer  Normal  Abnormal | 51  11 | 999  146 | Ref  1.48 | 0.75-2.90 | 0.26 |
| Hemoglobin  Normal  Low | 58  4 | 1067  78 | Ref  0.94 | 0.33-2.67 | 0.91 |
| Albumin | 42.0±3.1 | 43.0±3.1 | 0.94 | 0.87-1.01 | 0.08 |
| Liver function  Normal  Abnormal | 56  6 | 1087  58 | Ref  2.01 | 0.83-4.85 | 0.12 |
| Uric Acid  Normal  Low  High | 46  2  14 | 996  24  125 | Ref  1.80  2.43 | 0.41-7.48  1.30-4.54 | 0.43  0.01 |
| Serum creatinine  Normal  Low  High | 59  2  1 | 1072  43  30 | Ref  0.85  0.61 | 0.20-3.57  0.08-4.51 | 0.82  0.62 |
| Operation time | 123.1±50.8 | 93.9±33.2 | 1.02 | 1.01-1.02 | <0.001 |
| Blood loss  ≤100  >100 | 53  9 | 1116  29 | Ref  6.53 | 2.95-14.50 | <0.001 |
| Tumor marker  Normal  Abnormal | 43  19 | 908  237 | Ref  1.69 | 0.97-2.96 | 0.06 |
| previous history of pulmonary surgery  No  Yes | 60  2 | 1122  23 | Ref  1.63 | 0.37-7.06 | 0.52 |
| previous operation except pulmonary surgery  No  Yes | 43  19 | 799  346 | Ref  1.02 | 0.59-1.78 | 0.94 |
| Surgical site  Upper  Middle  Lower | 35  7  20 | 524  427  194 | Ref  0.54  0.70 | 0.24-1.24  0.40-1.23 | 0.14  0.22 |
| Pathology  benign  malignancy | 5  57 | 91  1054 | Ref  0.98 | 0.38-2.52 | 0.97 |
| Surgery  VATS  RATS | 42  20 | 749  396 | Ref  0.90 | 0.52-1.56 | 0.71 |
| NHSN risk index  0  1～2 | 35  27 | 968  177 | Ref  4.22 | 2.49-7.15 | <0.001 |

SSI, surgical site infection; MIS, minimally invasive surgery; VATS; video-assisted thoracic surgery; RATS, robotic-assisted thoracic surgery; BMI, body mass index; COPD, chronic obstructive pulmonary disease; ASA, American Society of Anesthesiologist; NHSN, National Healthcare Safety Network; OR, odd ratio; CI, confidence intervel.
